# Machine learning approaches demonstrate that protein structures carry information about their genetic coding

**DOI:** 10.1038/s41598-022-25874-z

**Published:** 2022-12-20

**Authors:** Linor Ackerman-Schraier, Aviv A. Rosenberg, Ailie Marx, Alex M. Bronstein

**Affiliations:** The Henry & Marilyn Taub Faculty of Computer Science, Technion – Israel Institute of Technology, 3200003 Haifa, Israel

**Keywords:** Protein folding, Computational science, Computer science

## Abstract

Synonymous codons translate into the same amino acid. Although the identity of synonymous codons is often considered inconsequential to the final protein structure, there is mounting evidence for an association between the two. Our study examined this association using regression and classification models, finding that codon sequences predict protein backbone dihedral angles with a lower error than amino acid sequences, and that models trained with true dihedral angles have better classification of synonymous codons given structural information than models trained with random dihedral angles. Using this classification approach, we investigated local codon–codon dependencies and tested whether synonymous codon identity can be predicted more accurately from codon context than amino acid context alone, and most specifically which codon context position carries the most predictive power.

## Introduction

Synthesis of proteins by translation is a fundamental cellular process, occurring in the ribosome, where amino acids are sequentially added to a growing chain according to the mRNA genetic instructions. The standard genetic code consists of 61 codons (and 3 dedicated as stop signals) that map uniquely to one of the 20 amino acids as part of a highly conserved, universal code. All but two amino acids are encoded by multiple, synonymous, codons. Although synonymous codon usage is mostly thought to be inconsequential to protein structure, some studies have suggested otherwise. Experiments have shown that codon usage can affect the structure and stability of mRNA and translation rate, which may in turn affect the structure of the resulting protein^1–7^. However the mechanisms by and the extent to which synonymous coding affects protein structure is still far from being fully understood.

We have recently uncovered a direct association between codon identity and the backbone dihedral angles $\phi$ and $\psi$, of the encoded amino acid, by comparing dihedral angle distributions of synonymous codons^8^. To determine if this association is detectable using other approaches, we apply regression based on deep neural networks (DNNs) and classification models based on gradient-boosted trees (XGB) and support-vector classifiers (SVCs) to determine: (i) whether protein structure prediction may be improved by using the genetic, rather than amino acid, sequence; (ii) whether the codon from which an amino acid was translated may be predicted given the final protein structure. Crucially, these questions need not assume a causal direction of the codon–structure association; that the association exists is, in principle, enough for these predictive tasks to be plausible. Adapting the structure–codon classification task to investigate codon–codon relations, we also sought to determine (iii) whether synonymous codon identity can be predicted from codon context better than from amino acid context, and if so, which context positions convey most predictive power.

Despite the considerable volume of research into protein structure prediction over the past half a century^9–11^, to the best of our knowledge, DNNs have not yet been used to probe for synonymous codon information in very local protein structure. Methods have been developed to predict the backbone dihedral angles as continuous labels^12–20^, with the majority relying on DNNs and their ensembles^19,21^. Most methods use position specific scoring matrices (PSSM) produced by PSI-BLAST^22^ as the input features^15–17,19,20^ and some additionally include the seven physicochemical properties (7PCP)^15–17,19,20,23^. To capture local structure around a given amino acid, sliding windows of varying sizes are commonly used^15–17,20^.

Here we show that using true codon information in order to predict backbone dihedral angles results in a statistically significant reduction in prediction error when compared with predictions based on random synonymous codons assignment of synonymous codons for each amino acid. We further show that providing the dihedral angles facilitates more accurate prediction of the true synonymous codon that was used to encode a specific amino acid. Both results provide additional evidence for the existence of a statistical dependence between synonymous genetic coding and local backbone structure. Finally, we demonstrate that codon usage at a focal position is significantly associated with the identities of synonymous codons around that position.

## Results

**Figure 1:**
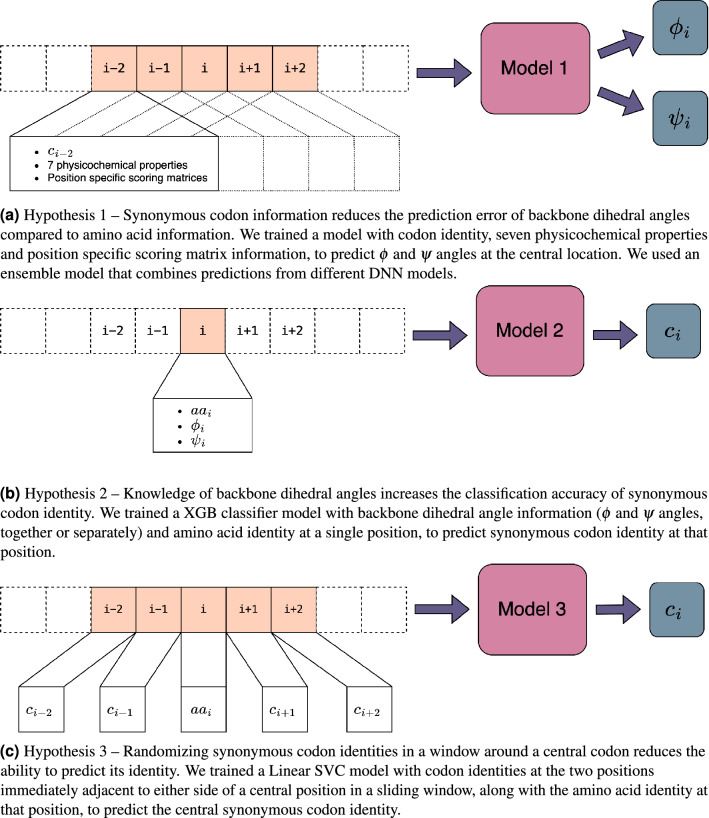
Schematic of model inputs and outputs for each hypothesis. Codon is indicated by *c* and amino acid is indicated by *aa*. Orange shading highlights the positions used as inputs. Only windows where all five positions have the same DSSP annotation were used and only H and E were considered, separately, in this work.

To determine (i) whether codon sequences have lower $\phi$ and $\psi$ prediction errors than the amino acid sequences, (ii) whether synonymous codon identity can be recognised from structural data; and (iii) whether synonymous codon identity prediction can be improved by giving codon context, we applied regression (Fig. 1a) and classification (Fig. 1b,c) models to local sequences in protein structures (Fig. 1). For the regression task we employed DNNs. Since these classification tasks have not been previously studied, we empirically compared DNN, SVC and XGB models. For Hypothesis 2, we selected XGB, and for Hypothesis 3, we selected SVC, which yielded better performance than the other models. Following the methodology of our recent distribution analysis of synonymous coding and backbone dihedral angle preferences^8^, we used only high resolution (better than 1.8Å) structures for which codons could be reliably assigned. Since we are using dihedral angles to describe the structure of the protein backbone, we included only structures where the electron density defines the position of these atoms well. Only windows where all five positions have the same DSSP annotation were used and only H (amino acids in $\alpha$ helices and referred to in this work as belonging to the $\alpha$ mode) and E (amino acids in $\beta$ strands and referred to in this work as belonging to the $\beta$ mode) were considered in this work. Additionally, $\alpha$ and $\beta$ modes were analyzed separately to control for known codon preference differences between these secondary structures and to facilitate the detection of coding information in very local structural contexts^24^.

It is important to note that unlike most other prediction tasks involving protein structures, we are limited here in terms of data since we would like to explore questions regarding synonymous codon usage, and the genetic sequences used to express the proteins are currently not annotated in the Protein Data Bank or other major structural databases. The implication is that we use only a small subset of PDB structures for which we can reliably assign codons, namely those structures resulting from sequences which are less likely to have been codon optimised. Furthermore, there is no gold standard with which to compare our results. To address this limitation, we include a control experiment to provide a baseline for assessing the significance of results obtained in testing each hypothesis.

### Hypothesis 1 (codon–structure): synonymous codon sequence information predicts protein backbone dihedral angles with lower error than amino acid sequence information

State-of-the-art dihedral angle prediction models use an amino acid sequence as their input. In order to probe whether the codon sequence carries additional information about the local structure, we trained a model which uses codons to predict protein backbone, $\phi$ and $\psi$, dihedral angles (Fig. 1a). To test this model against the null hypothesis that synonymous codons are not associated with differences in local structure, we evaluated the model once using true codon information on the test set and once using random synonymous codon information as a control. The random control codons were generated by random sampling of the synonymous codons from a re-normalized prior distribution of the codons after removing the true codon, as described in more detail in the “Methods”. It is important to note that the true codon was not used in the control sequences except in the case of amino acids encoded by a single codon. Our models utilized a similar architecture to those used in state-of-the-art dihedral angle prediction models, such as SPIDER^25^.

Comparison of the model errors showed that for the $\beta$ mode, the model predicted $\phi$ and $\psi$ with a statistically significantly lower error using the true codons compared to random synonymous codons, thus rejecting the null hypothesis (Table 1). True codon information did not improve prediction of $\phi$ or $\psi$ in a statistically significant manner for the $\alpha$ mode. This result is expected, since the $\alpha$ mode is more rigid in structure than the $\beta$ mode and so less likely to accommodate small, local structural variability^26,27^. It also agrees with our previous findings, that many synonymous codons have significantly different dihedral angle distributions in the $\beta$ mode, but none were observed in the $\alpha$ mode^8^. Table 1 also shows that the $\phi$ mean absolute error (MAE) is lower than for $\psi$ and that the error improvement using true codons is greater for $\phi$, in both $\beta$ and $\alpha$ modes. Previous works have also demonstrated that the prediction of $\phi$ is achieved with a lower error than $\psi$^28^. We demonstrate in Supplementary Fig. S1 that the angle estimation error obtained from our model is practically unbiased, with the mode, median, and mean being approximately zero degrees. The errors also show relatively low skewness.

**Table 1:** True synonymous codon information reduces the prediction error of backbone dihedral angles.

Secondary structure	Error measure	Error using true codons	Error using random codons	Error improvement using true codons	*p* value
$\beta$ mode	$\phi$ MAE	**19.5° ± 21.5°**	**19.6° ± 21.4°**	**0.14° ± 1.47°**	< 0.001*
	$\psi$ MAE	**21.2° ± 34.8°**	**21.2° ± 34.8°**	**0.05° ± 1.81°**	0.02*
$\alpha$ mode	$\phi$ MAE	4.1° ± 6.0°	4.1° ± 6.0°	8*E*−4° ± 0.21°	0.6
	$\psi$ MAE	4.3° ± 6.0°	4.3° ± 6.0°	4*E*−4° ± 0.21°	0.4

Significant results (*p* value $<0.05$) are indicated with *.

Significant values are in bold.

### Hypothesis 2 (structure–codon): protein backbone dihedral angle information improves synonymous codon identity classification accuracy

Having shown that synonymous codon identity facilitates better prediction of dihedral angles, we wanted to explore whether structural information, namely the configuration of dihedral angles, can improve the accuracy of predicting the synonymous codon used to translate a given amino acid. In order to design a framework which can test against the null hypothesis that dihedral angle information will not improve the accuracy of codon prediction, we trained a model to predict synonymous codon identity using the backbone dihedral angles and amino acid identity as the input. A comparison of the model’s accuracy was conducted using true structural information and random, control, structural information. The control structural information was derived from the same distribution as the true structural information but independent of codon identity. To that end, we re-sampled the structural data in order to obtain a balanced set of examples with the same number of examples for each codon (for more details, see “Methods”).

Table 2 shows the accuracy of synonymous codon classification when using true as compared to random $\phi$ and $\psi$ angles, together or separately, as the input. The accuracy of synonymous codon classification was significantly improved in both the $\alpha$ and the $\beta$ modes, when using the dihedral angles either together or individually. The percentage of degradation of the true compared to the control angles in the $\alpha$ mode was lower than those in the $\beta$ mode. only knowledge of the $\phi$ angle significantly improved codon classification Consistent with the conclusions of our first hypothesis, we find that the $\beta$ mode and $\phi$ distributions are more strongly associated with synonymous coding information compared to the $\alpha$ mode. or $\psi$. Since it has been shown that amino acid families grouped by their number of synonymous codons show distinctive patterns of codon biases and are under different selective constraints we ran the analysis for each of these families separately (2, 3, 4, and 6 codon amino acid families). The results shown in Supplementary Table S1 indicate that the 6 codon family has the best predictive accuracy using the true dihedral angles relative to the control.

**Table 2:** Structural information improves the classification accuracy of synonymous codon identity.

Secondary structure	Dihedral angle input	Accuracy using true angles	Accuracy using random angles	Accuracy degradation using random angles	% Accuracy degradation using random angles	*p* value
$\beta$ mode	$\phi$ and $\psi$	0.38	0.30	0.08	20.7	< 0.001*
	$\phi$	0.38	0.30	0.07	19.6	< 0.001*
	$\psi$	0.36	0.30	0.05	15.2	< 0.001*
$\alpha$ mode	$\phi$ and $\psi$	0.36	0.32	0.04	10.6	< 0.001*
	$\phi$	0.34	0.32	0.03	7.3	< 0.001*
	$\psi$	0.35	0.32	0.03	7.5	< 0.001*

Significant results (*p* value $<0.05$) are indicated with *.

### Hypothesis 3 (codon–codon): codon context information improves the accuracy of synonymous codon identity prediction

The finding that local structural information aids classification of synonymous codon identity suggests that the classification approach can be useful in investigating other codon usage associations in local protein structure. Here we adapt the model above to probe codon–codon associations in local protein structures.

In order to test whether the identity of a central synonymous codon is associated with the local codon sequence, we trained a model using only the codon sequence as an input, with the task of predicting the central codon’s identity, and tested the null hypothesis that randomizing synonymous codon identities around the central codon will not affect the accuracy in predicting that central codon. More specifically, we tested the importance of specific locations either individually or grouped. In order to accomplish this, the model was evaluated once by using the true codons in all positions, and again in a control experiment by randomizing synonymous codon identity at the positions of interest. By using random synonymous codons at different positions, we were able to analyze how synonymous codon usage at particular positions affects prediction accuracy.

**Figure 2:**
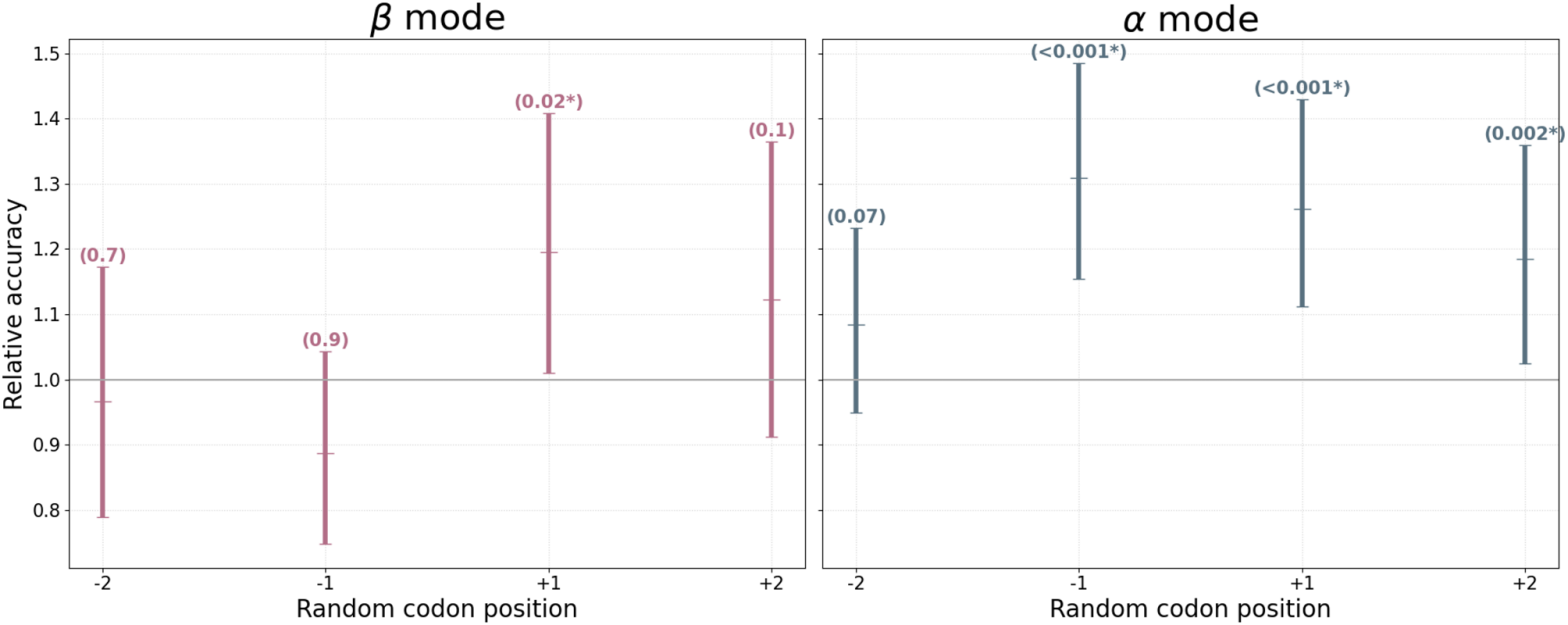
The effect of randomizing synonymous codon identity at different positions, on the prediction accuracy of the synonymous codon identity at the central position. The relative codon assignment accuracy is the comparison between the accuracy using true and random synonymous codons and it is expected that this ratio will be 1 if true and random synonymous codons have equal prediction power, as suggested by the null hypothesis. Mean and $95\%$ confidence intervals were estimated using bootstrapping: the original test set of size $N_{\alpha }=9575$ and $N_{\beta }=3378$ was subsampled with replacement to form 10, 000 independent samples of size $M_{\alpha }=6000$ and $M_{\beta }=3000$ for the $\alpha$ and $\beta$ mode respectively. The corresponding *p* values for rejecting the null hypothesis are listed in parenthesis, and the calculation of the *p* values is explained in greater detail in the “Methods”.

Our results show two main trends. Firstly the codon–codon dependence decays with distance from the central codon, and secondly, the existence of asymmetry in the extent to which adjacent coding information improves the prediction accuracy of a central codon. Figure 2 and Table 3 shows that in the $\beta$ mode, the prediction accuracy of a central codon is most significantly affected by the identity of the codon that is translated immediately after that codon (position $+1$ in the sequence window). Furthermore, these results show that in the $\alpha$ mode all of the positions, except position $-2$, statistically significantly affect the accuracy of predicting the central codon. Although in the $\alpha$ mode we see more positions with a significant association with the focal position compared to the $\beta$ mode, Table 3 shows similar percentages of accuracy degradation in both modes. The smaller *p* values in the $\alpha$ mode are likely the consequence of the larger sample size, since larger sample sizes generally increase the probability of finding a significant relationship^29^.

The detection of codon–codon associations is expected and the first observable trend, that this dependence decays with distance from the central codon, is also not surprising. Codon pair usage bias has been long recognised in *Escherichia coli*^30^ and more recently in human disease^31,32^. It has been suggested that codon translation efficiency in *E. coli* is modulated by adjacent single nucleotides^33^, that codon pair order significantly affects translation speed^34^, and more recently the case for a genetic code as a triplet of codons has been argued^35^. Studies have also shown that certain synonymous codons have preferences in transitions between secondary structures and coils^24,36^.

The second observable trend in our results is the existence of asymmetry in the extent to which adjacent coding information improves the prediction accuracy of a central codon. In our recent work^8^, we investigated the expected and observed number of codons in three locations of the $\beta$ mode (first two, last two and all middle residues of the $\beta$ strands) and found that there are some codons for which abundance at a particular location is significantly different ($p<0.05$) from the abundance observed in the full $\beta$ mode, which could explain the asymmetry we observe in these results. In a preliminary work we carried out the same analysis on a larger data set including all bacterial proteins expressed in *E. coli* and observe that the asymmetry around the central position remains but is less prominent than in this data set of only *E. coli* proteins expressed in *E. coli* (results not shown). The existence of the phenomenon in a larger data set supports our conclusions and the lesser prominence may be explained by the fact that codon abundances and codon pair biases vary from species to species.

**Table 3:** Codon context information improves the prediction accuracy of the central codon.

Secondary structure	Random codon positions	Accuracy	Accuracy degradation using random codons	% Accuracy degradation using random codons	*p* value
$\beta$ mode	[] - **All true codons**	0.46	−	−	−
	[$-2$, $-1$, $+1$, $+2$]	**0.43**	**0.02**	**5.4**	0.01*
	[$-1$, $+1$]	0.45	0.01	2.6	0.1
	[$-2$, $+2$]	0.45	0.005	1.1	0.1
	[$-2$, $-1$]	0.46	0.003	0.6	0.4
	[$+1$, $+2$]	**0.44**	**0.02**	**4.0**	0.02*
	[$-2$ ,$+1$, $+2$]	**0.43**	**0.03**	**5.9**	0.001*
	[$-2$, $-1$, $+2$]	0.46	0.0	− 0.2	0.6
	[$+1$]	**0.44**	**0.02**	**3.5**	0.02*
	[$-1$]	0.47	− 0.01	− 2.4	0.9
	[$-2$]	0.46	0.0	− 0.4	0.7
	[$+2$]	0.45	0.01	1.5	0.1
$\alpha$ mode	[] - **All true codons**	0.47	−	−	−
	[$-2$, $-1$, $+1$, $+2$]	**0.42**	**0.05**	**10.7**	< 0.001*
	[$-1$, $+1$]	**0.43**	**0.04**	**8.7**	< 0.001*
	[$-2$, $+2$]	**0.46**	**0.01**	**2.3**	0.01*
	[$-2$, $-1$]	**0.45**	**0.02**	**4.9**	< 0.001*
	[$+1$, $+2$]	**0.44**	**0.02**	**5.1**	< 0.001*
	[$-2$, $+1$, $+2$]	**0.44**	**0.03**	**6.4**	< 0.001*
	[$-2$, $-1$, $+2$]	**0.45**	**0.02**	**4.9**	< 0.001*
	[$+1$]	**0.45**	**0.02**	**4.2**	< 0.001*
	[$-1$]	**0.45**	**0.02**	**4.7**	< 0.001*
	[$-2$]	0.46	0.01	1.3	0.07
	[$+2$]	**0.46**	**0.01**	**2.3**	0.002*

The accuracy with which a central codon is predicted was tested when different combinations of context positions were provided with random synonymous codon information. Random codon position indices are arranged according to the central codon which is being predicted. An empty set of random positions corresponds to using true codons in all the positions and this serves as the baseline for calculating accuracy degradation. Significant results (*p* value $<0.05$) are indicated with *.

Significant values are in bold.

## Discussion

The machine learning models used in this work suggest the existence of non-trivial associations between synonymous codon usage and the structure of the translated amino acid. This cannot be attributed to synonymous codon-specific secondary structure preference, since we analyzed the $\alpha$ and $\beta$ modes separately. The analysis by secondary structure not only removes this potential bias but also reveals that the $\beta$ mode retains more coding information than the $\alpha$ mode; perhaps not surprising given that $\alpha$ helices fold into more rigid structures.

We note that the statistically significant error improvements in structure prediction using codons (Hypothesis 1) are very small, especially when compared to the size of the prediction error. It is worth recalling that our data set is of limited size, restricted to structures for which we have higher confidence in codon assignment. It may be that with more data, greater differences will be revealed. It is also worth noting that for proteins having a single and well defined structure, the vast majority of our data set, codon information is likely only retained at specific locations. Further studies should investigate how codon information can be best paired with other features and for which protein classes or specific locations in a protein, it can be most effective. The dependence between local backbone structure and synonymous coding was more pronounced when assigning codons from structure (Hypothesis 2). There, in the $\beta$ mode, the prediction of the synonymous codon identity is improved by about $18\%$ when the dihedral angles are provided. We note that the absolute accuracy of predicting the codon identity using the true structural data is not high (less than 0.38 in all cases). This is not surprising especially for the set of well folded, globular proteins that compose the data used in this study since we expect that the codon association will be detectable only in some locations of the final structure.

The detection of codon–codon associations in hypothesis 3 is expected and the first observable trend, that this dependence decays with distance from the central codon, is also not surprising. Codon pair usage bias has been long recognised in *E. coli*^30^ and more recently in human disease^31,32^. It has been suggested that codon translation efficiency in *E. coli* is modulated by adjacent single nucleotides^33^, that codon pair order significantly affects translation speed^34^, and more recently the case for a genetic code as a triplet of codons has been argued^35^. Studies have also shown that certain synonymous codons have preferences in transitions between secondary structures and coils^24,36^.

The second observable trend in our results is the existence of asymmetry in the extent to which adjacent coding information improves the prediction accuracy of a central codon. In our recent work^8^, we investigated the expected and observed number of codons in three locations of the $\beta$ mode (first two, last two and all middle residues of the $\beta$ strands) and found that there are some codons for which abundance at a particular location is significantly different ($p<0.05$) from the abundance observed in the full $\beta$ mode, which could explain the asymmetry we observe in these results. In a preliminary work we carried out the same analysis on a larger data set including all bacterial proteins expressed in *E. coli* and observe that the asymmetry around the central position remains but is less prominent than in this data set of only *E. coli* proteins expressed in *E. coli* (results not shown). The existence of the phenomenon in a larger data set supports our conclusions and the lesser prominence may be explained by the fact that codon abundances and codon pair biases vary from species to species.

The major novelty and contribution of this work is in demonstrating that machine learning methods can detect significant local codon–structure and codon–codon associations. It is important to stress that the consistency of conclusions from our models for Hypothesis 1 and 2 is non trivial. We probed these hypotheses with a different type of model, prediction task, and control experiment; yet the conclusion that a synonymous codon-dihedral angle association exists for the $\beta$ mode remains consistent. We believe that our findings highlight the need for inclusion of genetic sequences in protein structure databases and that they will further promote the use of genetic sequences in protein structure prediction tasks.

## Methods

### Dataset

We used the same data set as for our previous work Rosenberg et. al^8^. Full details of data collection are given in the “Methods” section of that work and a brief summary below.

High-resolution X-ray crystal structures with resolution no worse than 1.8Å and $R_{\textrm{free}}$ no worse than 0.24 were collected from the PDB. Redundant chains, in terms of pairwise sequence similarity, were excluded, as well as chimeric chains for which there was more than a single Uniprot identifier. The $R_{\textrm{free}}-R$ value was less than or equal to 0.7 for all structures with the following exceptions, which were included in the final dataset; (i) two structures had a slightly higher value of 0.9 and (ii) fifteen structures reported only the $R_{\textrm{free}}$ and not the R value. Since the genetic sequences used to express each structure are not available in the PDB, a codon was assigned to each residue by aligning the amino acid sequence with genetic sequences cross-referenced from the structures’ corresponding Uniprot record. Residues with an ambiguous or missing codon assignment were discarded from analysis. To reduce the chance of inaccurate codon assignment due to the widespread practice of codon-optimization, only *E. coli* proteins expressed in *E. coli* were used.

### Data splitting based on secondary structure

This work considered, separately, only regions having the DSSP secondary structure assignment^37^ E ($\beta$ mode) or H ($\alpha$ mode). By empirical evaluation, we chose an overlapping sliding window of size five as effectively ensuring context dependence of assumed local conformations. We only included windows where all the amino acids in that window had the same, E or H, DSSP assignment.

### Handling imbalanced data in classification problems

In the classification tasks of Hypotheses 2 and 3, our labels were codons. Since synonymous codons have vastly different relative abundances, our training data was unbalanced. In order to avoid a sampling bias associated with this imbalance, we used an over-sampling approach. In Hypothesis 2, for each amino acid family, we denote the amino acids that belong to this family as $[aa_1,\ldots , aa_k]$, and the codons that encode those amino acids by $[c_{1_1},\ldots ,c_{1_{n_1}}, \ldots ,c_{k_1},\ldots ,c_{k_{n_k}}]$. We consider the codon $c_i$ among $[c_{1_1},\ldots ,c_{1_{n_1}}, \ldots ,c_{k_1},\ldots ,c_{k_{n_k}}]$ with the highest number of examples in the training set, denoted by *m*. We then over-sample the training data of that amino acid family from all codons except $c_i$ in order to ensure that every codon has exactly *m* examples in this family. In Hypothesis 3, for each amino acid, we denote the codons that encode that amino acid by $[c_1,\ldots , c_n]$, and among those codons, we consider the codon $c_i$ with the highest number of examples in the training set, denoted by *m*. We then over-sample the training data from all codons except $c_i$ in order to ensure that every codon has exactly *m* examples.

### Description of models used for each hypothesis

#### Hypothesis 1 (codon–structure)

- **Input features.** We use a sliding window of five amino acids in which for each amino acid we utilized the standard genetic information by encoding the codon identity in a 61-dimensional one-hot encoding vector (Fig. 1a). In addition, for each position, we input the seven physicochemical properties^38^ (7PCP) and 20 values obtained from the PSSM generated by three iterations of PSI-BLAST^22^ against the UniRef90 sequence database updated in April 2018.
- **Predicted outputs.** The model predicts the $\phi$ and $\psi$ angles directly. We handle the angles’ periodicity (− 180$^{\circ }$ to 180$^{\circ }$) using the tanh activation function in the last layer, which returns values in the range (− 1, 1) and re-scale them by multiplying those outputs by 180. The loss function is calculated on the re-scaled values.
- **Loss functions.** We use the Manhattan distance ($L_1$ norm) loss function. For each predicted angle, ${\hat{\phi }}$ and actual angle, $\phi$, we calculate the absolute error $AE = |{\hat{\phi }}-{\phi }|$. The Manhattan distance is the sum of the absolute error of $\phi$ and $\psi$, $|{\hat{\phi }}-{\phi }|+|{\hat{\psi }}-{\psi }|$.
- **Model architecture and implementation**
  - **Neural network architecture** We employed two different, fully connected deep neural networks (FCDNNs), one to the $\beta$ mode model ($\beta$ mode FCDNN) and the other to the $\alpha$ mode model ($\alpha$ mode FCDNN). The $\beta$ mode FCDNN had the corresponding layers: 440$\rightarrow$32/64/128$\rightarrow$2, while the $\alpha$ mode FCDNN had the corresponding layers: 440 $\rightarrow$16/32/64/128$\rightarrow$2. The activation function in the output layer was tanh and relu in the input and the hidden layers.
  - **Model Training.** Training was performed using a stochastic gradient descent (SGD) optimiser with momentum 0.9, and decaying learning rate starting at 0.01 and reduced by a factor of 0.5 each time 3 successive epochs produce no improvement in the validation loss, flattening at $10^{-15}$. The training was terminated if the validation loss functions did not improve in 10 consecutive epochs. The parameters were initialized using the xavier-uniform initialization.
  - **Ensemble model.** The predictions were made by an ensemble model which combines the predictions of 50 different FCDNNs. We trained each model on a random split of the training data, as follows: we randomly selected 40 Uniprot ids to define the PDB structures of the validation set, and the remaining Uniprot ids were used for defining the training set. As a result, 50 models were obtained, each trained on a different subset of the training data. The test set was kept aside and was the same for each model used to construct the ensemble model.
- **Construction of control synonymous codons**—As control codons, we used random false synonymous codons. In order to account for the different abundances of synonymous codons, we randomized a given codon $c_0$ with *n* synonymous codons $\{c_1, c_2, \ldots , c_n\}$. Let us denote by $\{p_{c_0}, p_{c_1}, p_{c_2}, \ldots , p_{c_n}\}$ the probabilities of all synonymous coding possibilities estimated from the codon frequencies in the training set. We re-normalized this prior distribution by removing the true codon $c_0$, meaning for each synonymous variant $i \in \{1, \ldots , n\}$, probability $p'_{c_i} = \frac{p_{c_i}}{1 - p_{c_0}}$ was assigned. Note that $\sum _{i=1}^{n} p'_{c_i} = 1$. Finally, we randomly selected a codon from $\{c_1, c_2, \ldots , c_n\}$ based on the distribution $\{p'_{c_1}, p'_{c_2}, \ldots , p'_{c_n}\}$, and this codon was be used as the input to the model in place of the true codon $c_0$. Since the process described above is performed on all codons in each input window, we obtained that the input to the model consists of random, false synonymous codons.
- **Analysis of the effect of codon sequence information on the prediction of protein dihedral angles.** We calculated two mean absolute error (MAE) measures for each of those evaluation methods: $\phi$ MAE and $\psi$ MAE. For each predicted angle, ${\hat{\phi }}$ and actual angle, $\phi$, the absolute error was calculated ${\textrm{AE}} = |{\hat{\phi }}-{\phi }|$, followed by averaging over all prediction. The *p* value w.r.t. the null hypothesis, that the codon information does not reduce the prediction error of backbone dihedral angles, was calculated by comparing the results obtained using the true and the random false synonymous codons using the one-sided paired *t*-test, separately for each of the two error measures ($\phi$ MAE and $\psi$ MAE), resulting in a total of two *p* values per model.

#### Hypothesis 2 (structure–codon)

- **Input features and predicted outputs.** For each window of five amino acids that share the same secondary structure, we focused on the central amino acid. Thus, the model only considered the central amino acid, and no context was taken into account. As the input to the model, the amino acid information and the dihedral angle of its backbone in the given window were provided. The amino acid was encoded with a 20-dimensional one-hot encoding vector and the backbone dihedral angle is normalized to be in the range $[-1, +1]$ by dividing each angle by 180. Our experiment included providing the $\phi$ and $\psi$ angles together or separately. Given the input mentioned above, the model predicted a single output, the codon for the central amino acid.
- **Model architecture and implementation.** We utilized the xgboost implementation of the XGB Classifier. We handled the multiclass classification using the softmax objective, which returns the predicted class. On the basis of the evaluation set, the default parameters of the XGB classifier were selected.
- **Construction of control structural information.** We used random backbone dihedral angles as control structural information. To randomize the angle of a given the *i*-th amino acid, let us refer to $aa_i$, $\phi _i$, $\psi _i$ and $c_i$ as their respective amino acid, backbone angles, and codon. First, we filter all of the training set examples to find those that come from the same amino acid family as our example $aa_i$. Over-sampling is then used to balance this filtered set so that we have the same number of examples of each codon encoding $aa_i$ in this amino acid family. We then filter the balanced set of examples to have only examples of codons encoded by $aa_i$ and we randomly select the *j*-th example from among this balanced set of examples and use its backbone dihedral angles $\phi _j$ and $\psi _j$ as control angles, instead of the true backbone dihedral angles $\phi _i$ and $\psi _i$, and retain the target codon as $c_i$.
- **Analysis of the effect of protein dihedral angles on synonymous codon identity classification.** To compare the performance of using the true and random backbone dihedral angles, the accuracy measure was used. Each example from the test set was evaluated on the pre-trained classifier. We denote the classification result (True or False) using the true backbone dihedral angles as $r_1$ and using the random backbone dihedral angles as $r_2$ where $r_1, r_2 \in \{T, F\}$. For convenience, we will denote the joint classification outcome of the two evaluation methods as $r_{{r_1},{r_2}}$, where $r_{{r_1},{r_2}} \in \{r_{T,T}, r_{F,F}, r_{T,F}, r_{F,T}\}$. Over all the test set, we sum the number of times we obtain each classification outcome denoting as $\{N_{T,T}, N_{F,F}, N_{T,F}, N_{F,T}\}$ respectively. To calculate the accuracy of using the true vs. random backbone dihedral angles, we compared $N_{T,F}, N_{F,T}$. $N_{T,F}$ indicates the number of times classification error was incurred when randomizing the backbone dihedral angles but not incurred when using the true backbone dihedral angles and $N_{F,T}$ indicates the number of times classification error was incurred when using true backbone dihedral angles but not incurred when randomizing the backbone dihedral angles. Relative codon assignment accuracy is calculated as $\frac{N_{T,F}}{N_{F,T}}$. The exact one-sided binomial test was used to calculate the *p* value to determine the significance of the true backbone dihedral angles using $N_{T,F}$ being superior to random angles results using $N_{F,T}$ in the classification task.

#### Hypothesis 3 (codon–codon)

- **Input features and predicted outputs**. For each position in the window of five, we attempted to predict from the context of the codons $[c_{-2}, c_{-1}, c_{+1}, c_{+2}]$ the identity of the central codon $c_0$, out of the synonymous variants encoding amino acid $aa_0$. In order to use genetic information for classification, we encoded the context codons of each amino acid in the window in a 61-dimensional one-hot encoded vector. The amino acid identity of the central amino acid was encoded with a 20-dimensional one-hot encoding vector.
- **Model architecture and implementation**—We utilized the sklearn implementation of Linear SVC and applied a one-vs.-all strategy to deal with multiclass cases. The regularization parameter (C) was set to 0.035.
- **Construction of control synonymous codons.** The construction of control synonymous codons was performed as in Hypothesis 1.
- **Analysis of the impact of codon context on the classification of central synonymous codon identity.** We calculated the accuracy of using true and control synonymous codons to compare their performance using the relative codon assignment accuracy. To determine whether genetic information is more preferable to synonymous random codons, the *p* value was calculated as Hypothesis 2, using the exact one-sided binomial test.

## Supplementary Information

Supplementary Information.

## Data Availability

The data used in this study are freely available^39^.

## Code availability

The code implementing the described data collection and analysis methods has been deposited in the e Zenodo repository [https://doi.org/10.5281/zenodo.6814322]. Code is available under restricted access conditioned on user identification and agreement to academic use license.
