# Supplementary material for: Machine learning approaches demonstrate that protein structures carry information about their genetic coding
Source: Sci Rep. 2022 Dec 20;12:21968. doi: 10.1038/s41598-022-25874-z (PMC9767929; doi:10.1038/s41598-022-25874-z)
Supplement: Supplementary file 1 — Supplementary Information. [file 41598_2022_25874_MOESM1_ESM.pdf]

Machine learning approaches demonstrate that protein structures carry information about their genetic coding

Linor Ackerman-Schraier, Aviv A. Rosenberg, Ailie Marx, Alex M. Bronstein

Supplementary Figures

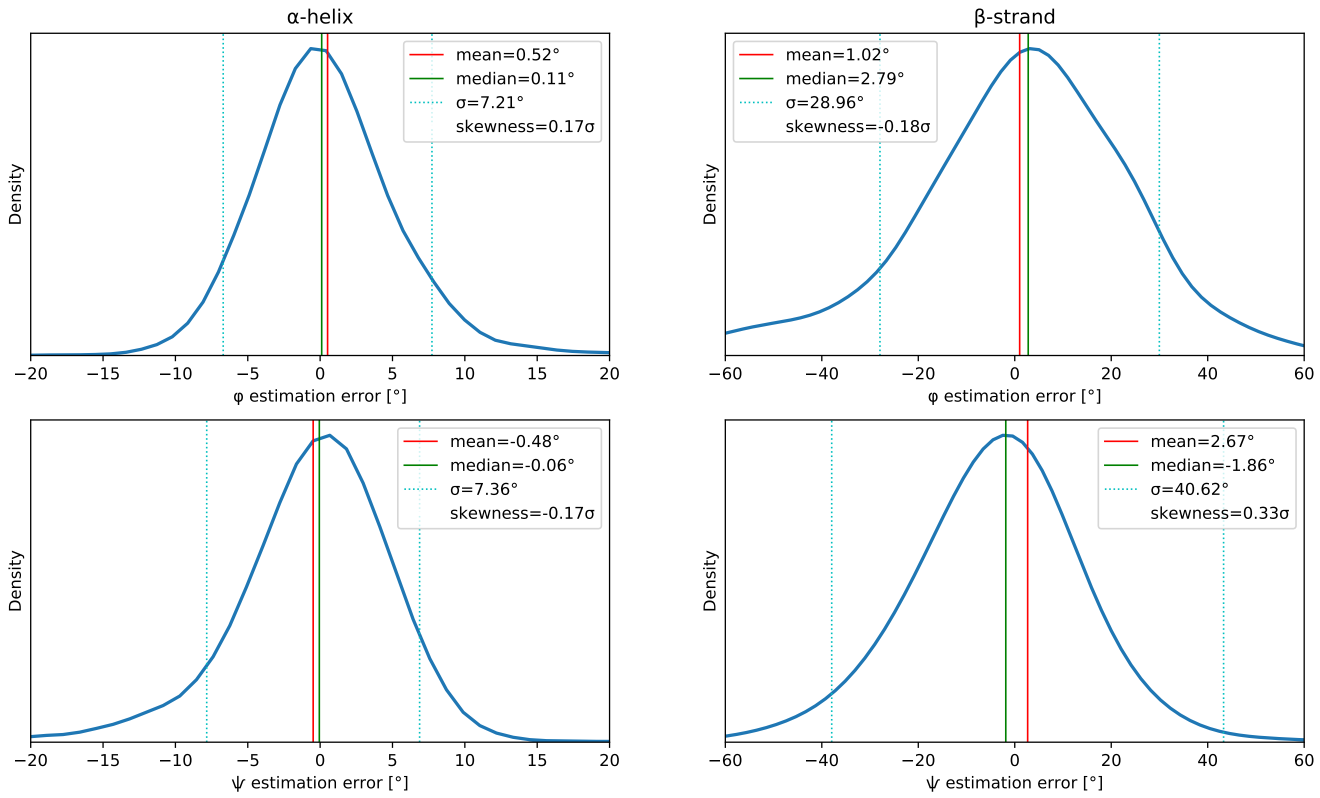

Figure S1. The angle estimation error for hypothesis 1 results. Visualization of the errors, opted for the following KDE plots. The plots include basic statistics such as mean, median, standard deviation and skewness (the latter estimated as Pearson's median skewness, $3*(mean - median)$, in units of sigma).

Supplementary Tables

**Dihedral angle input**	**Amino acid Family**	**Accuracy of random guess**	**Accuracy using true angles**	**Accuracy using random angles**	**Accuracy degradation using random angles**	**% Accuracy degradation using random angles**	**p value**
ϕ and ψ	2 codons	0.5	**0.52**	**0.49**	**0.03**	**6.2%**	**0.005***
	3 codons	0.33	**0.40**	**0.34**	**0.06**	**13.9%**	**0.02***
	4 codons	0.25	0.27	0.26	0.01	2.2%	0.3
	6 codons	0.17	**0.23**	**0.15**	**0.08**	**34.7%**	**<0.001***
	Combined		**0.36**	**0.32**	**0.04**	**10.6%**	**<0.001***
ϕ	2 codons	0.5	0.51	0.50	0.01	2.2%	0.2
	3 codons	0.33	0.36	0.34	0.02	6.3%	0.2
	4 codons	0.25	**0.27**	**0.24**	**0.02**	**8.6%**	**0.02***
	6 codons	0.17	**0.19**	**0.14**	**0.05**	**25.0%**	**<0.001***
	Combined		**0.34**	**0.32**	**0.03**	**7.3%**	**<0.001***
ψ	2 codons	0.5	0.51	0.50	0.01	1.4%	0.3
	3 codons	0.33	**0.39**	**0.33**	**0.05**	**13.9%**	**0.02***
	4 codons	0.25	0.27	0.25	0.02	7.0%	0.1
	6 codons	0.17	**0.19**	**0.14**	**0.06**	**29.3%**	**<0.001***
	Combined		**0.35**	**0.32**	**0.03**	**7.5%**	**<0.001***

(a) Secondary structure - α mode.

**Dihedral angle input**	**Amino acid Family**	**Accuracy of random guess**	**Accuracy using true angles**	**Accuracy using random angles**	**Accuracy degradation using random angles**	**% Accuracy degradation using random angles**	**p value**
ϕ and ψ	2 codons	0.5	**0.54**	**0.46**	**0.08**	**14.3%**	**<0.001***
	3 codons	0.33	**0.41**	**0.30**	**0.11**	**26.5%**	**0.003***
	4 codons	0.25	**0.29**	**0.25**	**0.05**	**16.7%**	**0.005***
	6 codons	0.17	**0.28**	**0.16**	**0.12**	**42.8%**	**<0.001***
	Combined		**0.38**	**0.30**	**0.08**	**20.7%**	**<0.001***
ϕ	2 codons	0.5	**0.55**	**0.46**	**0.09**	**16.0%**	**<0.001***
	3 codons	0.33	**0.44**	**0.30**	**0.14**	**32.1%**	**<0.001***
	4 codons	0.25	**0.28**	**0.25**	**0.04**	**13.4%**	**0.02***
	6 codons	0.17	**0.24**	**0.16**	**0.08**	**33.3%**	**<0.001***
	Combined		**0.38**	**0.30**	**0.07**	**19.6%**	**<0.001***
ψ	2 codons	0.5	0.49	0.46	0.03	5.9%	0.1
	3 codons	0.33	**0.45**	**0.33**	**0.12**	**27.0%**	**<0.001***
	4 codons	0.25	**0.29**	**0.24**	**0.05**	**18.2%**	**0.002***
	6 codons	0.17	**0.21**	**0.15**	**0.06**	**29.8%**	**0.002***
	Combined		**0.36**	**0.30**	**0.05**	**15.2%**	**<0.001***

(b) Secondary structure - β mode.

Table S1. Structural information improves the classification accuracy of synonymous codon identity. We employed the Benjamini-Hochberg correction with false discovery rate set to 0.05 and indicated significant results with *. The analysis is based on four types of amino acid families being encoded by: 2, 3, 4 and 6 codons. The combined results of all the families for a given dihedral angle input have been highlighted in pink for completeness.
